# Mechanistic insights into lethal hyper progressive disease induced by PD-L1 inhibitor in metastatic urothelial carcinoma

**DOI:** 10.1038/s41698-024-00707-6

**Published:** 2024-09-17

**Authors:** Kazuki Nishimura, Kiyoshi Takahara, Kazumasa Komura, Mitsuaki Ishida, Kensuke Hirosuna, Ryoichi Maenosono, Masahiko Ajiro, Moritoshi Sakamoto, Kengo Iwatsuki, Yuki Nakajima, Takuya Tsujino, Kohei Taniguchi, Tomohito Tanaka, Teruo Inamoto, Yoshinobu Hirose, Fumihito Ono, Yoichi Kondo, Akihide Yoshimi, Haruhito Azuma

**Affiliations:** 1 Department of Urology, Osaka Medical and Pharmaceutical University, Takatsuki City, Osaka Japan; 2 Division of Cancer RNA Research, National Cancer Center Research Institute, Chuo-Ku, Tokyo Japan; 3 Department of Urology, Fujita-Health University School of Medicine, Toyoake City, Aichi Japan; 4 Division of Translational Research, Osaka Medical and Pharmaceutical University, Takatsuki City, Osaka Japan; 5 Department of Pathology, Osaka Medical and Pharmaceutical University, Takatsuki City, Osaka Japan; 6 Department of Regenerative Science, Okayama University Graduate School of Medicine, Dentistry and Pharmaceutical Sciences, Okayama City, Okayama Japan; 7 Department of Anatomy and Cell Biology, Faculty of Medicine, Osaka Medical and Pharmaceutical University, Takatsuki, Osaka Japan

**Keywords:** Bladder cancer, Cancer microenvironment, Immunotherapy

## Abstract

Hyper progressive disease (HPD) is a paradoxical phenomenon characterized by accelerated tumor growth following treatment with immune checkpoint inhibitors. However, the pathogenic causality and its predictor remain unknown. We herein report a fatal case of HPD in a 50-year-old man with metastatic bladder cancer. He had achieved a complete response (CR) through chemoradiation therapy followed by twelve cycles of chemotherapy, maintaining CR for 24 months. Three weeks after initiating maintenance use of a PD-L1 inhibitor, avelumab, a massive amount of metastases developed, leading to the patient’s demise. Omics analysis, utilizing metastatic tissues obtained from an immediate autopsy, implied the contribution of M2 macrophages, TGF-β signaling, and interleukin-8 to HPD pathogenesis.

## Introduction

Bladder cancer (BC) ranked as the ninth most prevalent cancer in the world (sixth in men and seventeenth in women), with the incidence steadily rising each year^1,2^. BC is classified into non-muscle invasive BC (NMIBC) and muscle-invasive BC (MIBC) based on the presence or absence of muscle layer invasion. Despite curative therapy (such as cystectomy or a combination of curative chemotherapy, radiation, and maximal transurethral resection of bladder tumor (TUR-BT)) for high-risk NMIBC and MIBC, BC exhibits a high recurrence rate. For decades, platinum-based chemotherapy has served as the standard treatment for metastatic urothelial carcinoma (mUC), yielding a median survival of only 14–15 months^3,4^. Since 2017, immune checkpoint inhibitors (ICIs) have contributed to significant therapeutic advances in the treatment of mUC^5–7^. In the JAVELIN Bladder 100, an international open-label phase 3 trial, avelumab maintenance treatment targeting programmed death-ligand 1 (PD-L1) after the receipt of four to six cycles of platinum-based combination chemotherapy demonstrated significantly improved overall survival and progression-free survival compared to the best supportive care control group in advanced or metastatic UC^8^. Avelumab maintenance treatment has now established itself as the standard of care for mUC after achieving disease control with platinum chemotherapy. However, certain patient populations do not respond to this treatment. Several recent reports have described rare cases of hyper progressive disease (HPD) in response to ICIs. Although specific genetic alterations and/or tumor microenvironment (TME) are presumably associated with HPD, the mechanistic insights into this phenomenon remain largely unexplored.

Herein, we demonstrate a case of massive HPD that occurred immediately after the initiation of maintenance avelumab in a patient with mUC, resulting in a lethal outcome. Following full consent from the family, a pathological autopsy was performed, providing specimens of the metastatic lesions from the mesentery, iliopsoas muscle, para-aortic lymph node (para-aortic LN), and liver. To unveil the molecular feature of HPD, we conducted an omics analysis utilizing whole-exome sequencing (WES), RNA-sequencing (RNA-seq), and multiplex immunohistochemistry. ELISA was also performed on peripheral blood samples stored in our biobank for the cytokine array.

## Result

### Clinical case

**Fig. 1:**
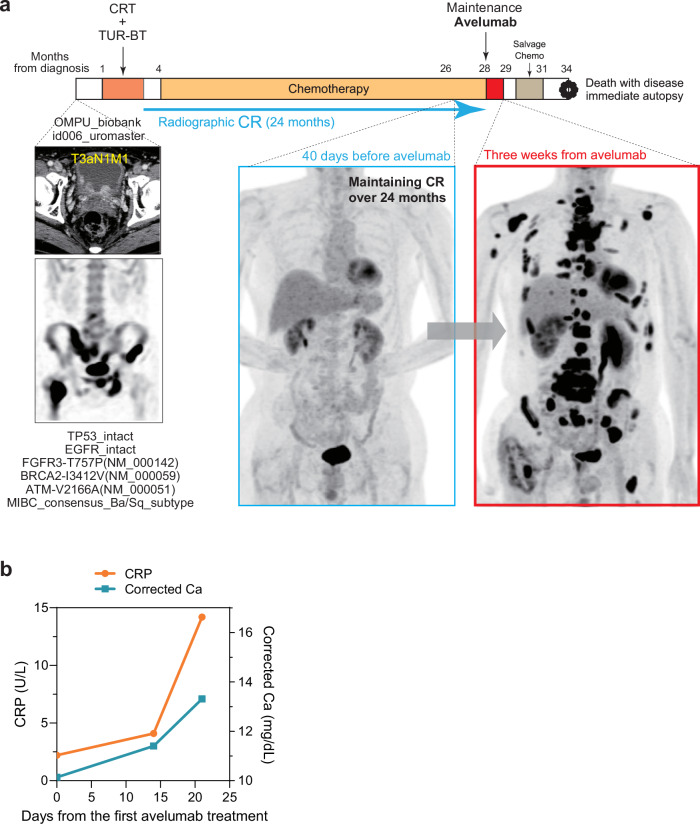
Clinical timeline of the patient. **a** Clinical course and radiographic images of this case along with representative pathogenetic information. **b** Changes in levels of C-reactive protein and corrected calcium concentration in peripheral blood after avelumab treatment. Day 0 corresponds to the day of the first avelumab administration.

The clinical course of the case is illustrated in Fig. 1a. A 50-year-old man presented to our affiliated hospital with gross hematuria in 2019, leading to the diagnosis of a bladder tumor. Maximal TUR-BT confirmed invasive urothelial carcinoma with no histological variant, high grade, positive lymph vascular invasion, and pT3a. WES and RNA-seq were conducted on the primary tumor as part of the OMPU-NCC Cancer Consortium Project^9,10^, which provided the following genetic information for this case, such as TP53_intact, EGFR_intact, FGFR3-T757P (NM_000142), BRCA2-I3412V (NM_000059), ATM-V2166A (NM_000051), and MIBC consensus Ba/Sq subtype^11^. Imaging studies, including computed tomography (CT) and bone scintigraphy, revealed regional lymph node and multiple pelvic bone metastases, leading to the diagnosis of pT3aN1M1. The patient underwent chemoradiation therapy, consisting of pelvic irradiation (whole pelvic external beam radiation therapy (EBRT): 2 Gy × 25 fractions, total 50 Gy) and GC chemotherapy (gemcitabine: 1000 mg/m² and cisplatin: 70 mg/m² in a 28-day cycle). Following the chemoradiation therapy, the patient exhibited an exceptional response, achieving complete response (CR). Thereafter, the patient received 12 cycles of GC chemotherapy over 24 months with intermittent drug holidays, maintaining CR in radiographic examinations.

Since maintenance avelumab therapy for mUC patients was approved in 2021, the patient transitioned to this maintenance treatment. While the initial administration showed no apparent adverse events, the patient reported mild physical discomfort during the second administration. Three weeks after the first avelumab treatment, the patient was urgently admitted to our hospital with pronounced general fatigue and severely elevated inflammatory response as well as hypercalcemia (Fig. 1b). Fluorodeoxyglucose-18F positron emission tomography (FDG-PET) revealed extensive metastases in the bones, lymph nodes, iliopsoas muscle, soft tissues, pancreas, and liver. Despite the administration of salvage chemotherapy using gemcitabine (1000 mg/m²) and paclitaxel (150 mg/m²) in a 21-day cycle, the patient’s general status rapidly deteriorated with no clinical response. The patient consequently succumbed to disease progression six months after the first dose of avelumab.

Given the rapid and atypical clinical course of the case, we obtained consent from the family and proceeded with a pathological autopsy. The autopsy yielded specimens from four metastatic lesions (mesentery, iliopsoas muscle, para-aortic LN, and liver). These, along with the pre-treatment primary bladder tumor, were subjected to WES, RNA-seq, and pathological investigation to delve into the molecular underpinnings of the HPD. Additionally, stored plasma samples stored in the OMPU-biobank (collected before and after avelumab treatment) for three patients treated with avelumab, including this case, were analyzed using ELISA to examine changes in cytokine levels.

### TGF-β signaling is activated in metastatic tumors

**Fig. 2:**
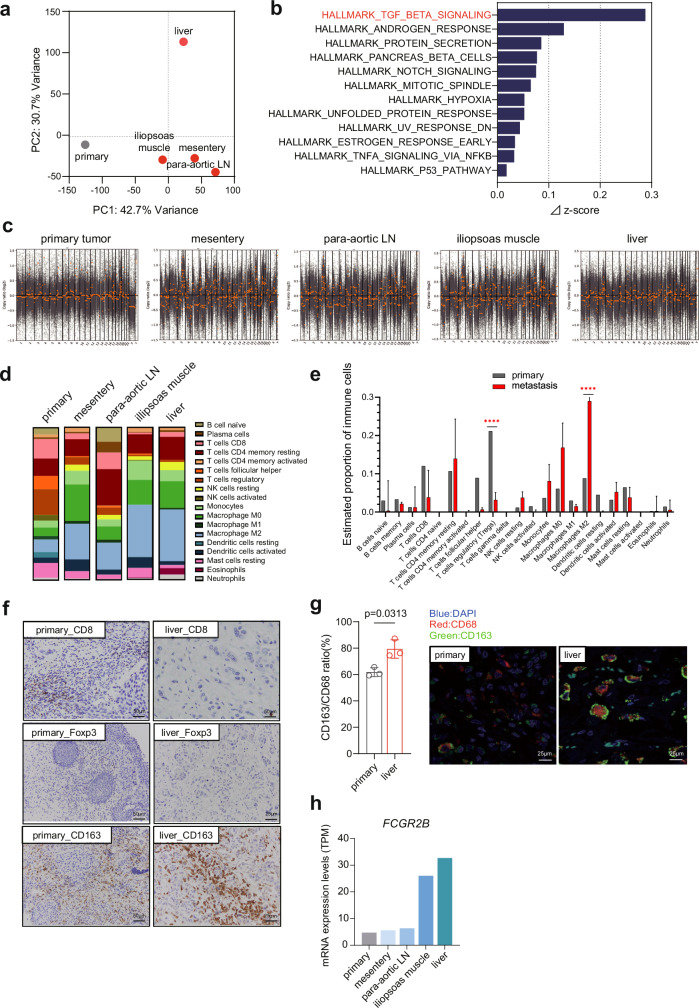
The analysis of next generation sequencing (NGS) results and pathological validation. **a** Principal component analysis for whole transcriptome data in primary and metastatic samples. **b** Activated gene sets in metastatic tumors compared to primary tumor in hallmark gene sets. ⊿z-score represents the difference in z-score between the average of metastatic tumors and primary tumor. **c** Genome-wide frequency plot of copy number alterations in primary and metastatic tumors. The log2 ratio for each of the genomic clones is plotted according to chromosome positions. **d** Estimated proportion of immune infiltration in primary and metastatic tumors using the CIBERSORTx. **e** The difference of immune infiltration between primary and metastatic tumors. Gray and red bar represent estimated proportion of immune cells in primary tumor and metastatic tumor, respectively. Error bars show the 95% confidence interval. *p* values were calculated by two-way ANOVA. *p* < 0.0001****. **f** Representative images of CD8, FOXP3 and CD163 immunohistochemistry in primary and liver metastatic tumors. **g** The ratio of CD163/CD68 positive cells detected by immunohistochemistry in primary and liver metastatic tumors (left) and representative multiple immunofluorescent staining (right). Nuclei, pan-macrophages, and M2-macrophages were stained blue, red, and green, respectively, using specific antibodies (shown in Methods). *P* value was calculated by Welch’s test. **h** FCGR2B mRNA expression levels in primary and metastatic tumors were shown in transcript per million (TPM).

The RNA-seq data from metastatic tumors provided us with the Ba/Sq consensus molecular subtype for these tumors, which was the same as that of the primary tumor. PCA analysis of primary and metastatic tumors revealed distinct gene expression patterns between the primary and metastatic tumors (Fig. 2a). Furthermore, our analysis suggested that liver metastases exhibit a unique gene expression pattern compared to other metastatic sites. To explore the activated gene signature in HPD, we compared gene expression levels between the primary tumor and metastatic tumors using RNA-seq data. We calculated the z-score for 50 hallmark gene sets in each specimen^12^. Comparing the z-score of the primary tumor with the mean values of metastatic samples, we found that 12 of the 50 gene sets were activated in metastatic tumors among 50 hallmark gene sets. Notably, TGF_BETA_SIGNALING was the most activated gene set in metastatic specimens, which was reported to act immune-suppressively in the TME and promote tumor progression especially in the late stage of cancer^13–15^ (Fig. 2b, Supplementary Data 1).

### CNA analysis and infiltration of M2 macrophage in TME of HPD

Subsequently, we assessed copy number alterations (CNAs) across the samples. Compared to the primary tumor, diverse CNAs, which were reported to be associated with poorer response to ICI^16^, were observed in the metastatic tumors, suggesting genetically heterogeneous evolutions within the metastatic tumor cells (Fig. 2c).

Next, we employed CIBERSORTx, a method of digital flow cytometry^17^, to reconstruct the composition of 22 types of immune cells within the tumor cells using bulk transcriptomic data (Fig. 2d). Among the 22 types of immune cells analyzed, we observed a significant increase in the ratio of M2 macrophages and a decrease in the ratio of regulatory T-cells (T-regs) in the metastatic lesions compared to the primary tumor (*p* < 0.0001) (Fig. 2e). These findings suggest the possibility of T-reg depletion and increased infiltration of M2 macrophages in the case of HPD. To validate this hypothesis, we conducted pathological examinations. Immunohistochemistry revealed no differences in FOXP3-positive cells or CD8-T cells, but there was a marked increase in CD163-positive cells in the metastatic lesions, especially in the liver metastasis, compared to the primary tumor (Fig. 2f). In addition, immunohistochemistry and multiplex immunofluorescent staining confirmed that the ratio of CD163/CD68-positive cells was significantly increased in the liver metastatic tumor (*p* = 0.0313) (Fig. 2g), suggesting the crucial role of M2 macrophage infiltration in the pathogenesis of HPD. In line with these findings, FCGR2B (FcγRIIB), a unique inhibitory Fcγ receptor expressed in macrophages^18,19^, was upregulated especially in the iliopsoas and liver metastases compared to the primary tumor (Fig. 2h).

### Elevation of plasma IL-8 level in HPD

**Fig. 3:**
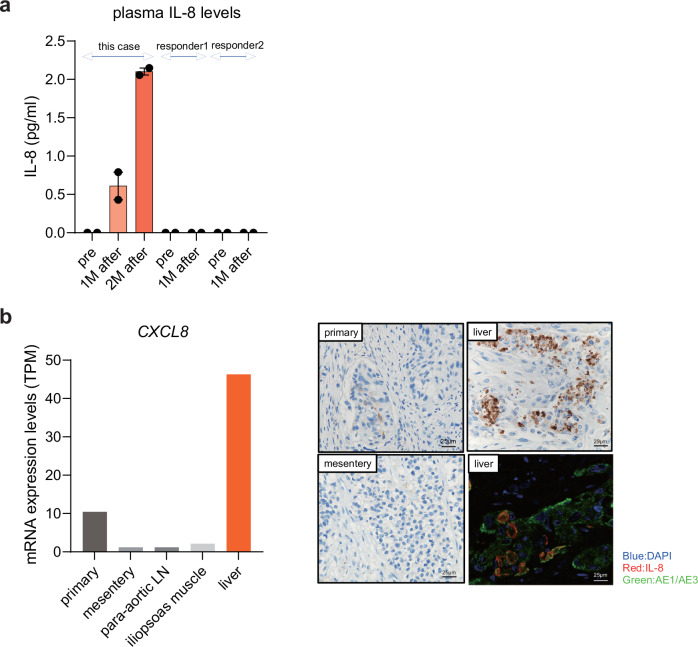
Plasma IL-8 level chronologically increased in the HPD case. **a** ELISA analysis of 19 cytokines was conducted on plasma samples from three patients treated with avelumab (including this case and two avelumab responders). The plasma samples were collected before avelumab treatment, and at one and two months after the first avelumab treatment. **b** Left: mRNA expression level (transcripts per million: TPM) analysis of CXCL8 (IL-8). Right: Representative IL-8 immunohistochemistry and multiple immunofluorescent staining of primary and metastatic (mesentery and liver) tumors. Nuclei, tumor cells, and IL-8 were stained blue, green, and red, respectively, using specific antibodies (shown in Methods).

We further conducted a ProcartaPlex immune assay® to quantitate various cytokines in paired plasma samples (pre- and post-avelumab treatment) of three patients (the present HPD case and two avelumab responders). Among the measured 19 cytokines, we identified plasma interleukin-8 (IL-8) as a unique and potential key cytokine in HPD, showing chronological elevation after avelumab treatment exclusively in this HPD case (Fig. 3a, Supplementary Data 2). Strikingly, we discovered that IL-8 was primarily produced by liver metastatic tumor cells (Fig. 3b). These findings suggest the important role of IL-8 in the process of HPD pathogenesis.

## Discussion

HPD is an unexpectedly rapid progression of disease in patients receiving immunotherapy and is one of the characteristic and critical adverse events in ICI treatment. We believe this to be the first reported case of HPD in mUC treated with maintenance use of avelumab, resulting in rapid tumor growth and ultimately death in an extremely short term. Although two or more metastatic areas and advanced age (≥65) have been reported to be associated with HPD as clinical variables so far^20,21^, our case did not meet these variables. In the context of TME, although a decrease in the number of tumor-infiltrating lymphocytes has been reported in the cases of HPD, the present case exhibited limited CD8 + T-cells in both the primary and metastatic tumors (Fig. 2f). A recent report suggested that the interaction between M2-like macrophages and nivolumab, a PD-1 inhibitor, through the Fc-Fcγ receptor is associated with HPD^22^. Avelumab is an anti–PD-L1 human immunoglobulin G1 (IgG1) monoclonal antibody (mAb) with a wild-type Fc domain, enabling it to interact with and activate various human Fcγ receptor signaling pathways. This is unlike other anti-PD-L1 agents, such as atezolizumab and durvalumab, which have mutant Fc domains. FcγRIIB is the unique inhibitory Fcγ receptor that directly antagonizes the immunostimulatory intracellular signals of activating Fcγ receptors. It has been reported to be significantly upregulated on dendritic cells and macrophages in the TME^18,19^. In a preclinical model, combination therapy with avelumab and an anti-human FcγRIIB–blocking mAb significantly increased the therapeutic effect compared to avelumab monotherapy, indicating that FcγRIIB inhibits the antitumor activity of avelumab in the TME^19^. Our examination revealed M2 macrophage infiltration in metastatic lesions compared to the primary tumor. Intriguingly, FCGR2B (FcγRIIB) mRNA expression levels were especially increased in iliopsoas muscle and liver metastatic tumors (Fig. 2h), potentially emphasizing the role of M2 macrophages in the pathogenic mechanism of HPD. It is also known that the differentiation of tumor-associated macrophages (TAMs) from monocytes is induced via IL-8 activation^23^ and increased serum IL-8 levels during ICI treatment are related to worse overall survival in mUC and metastatic renal cell carcinoma^24^. Moreover, we identified the activation of TGF-β signaling in metastatic tumors. TGF-β has also been reported to induce the polarization of M2-like macrophage^25^, which, in turn, are involved in tissue repair by secreting growth factors such as TGF-β^26,27^. Since the combination of ICI and TGF-β blocking antibodies has been shown to reduce TGF-β signaling and induce anti-tumor immunity, tumor regression in many preclinical models^15,28–31^, this combination therapy might be effective in preventing HPD.

To date, several pathogenic mechanisms and biomarkers related to HPD have been reported. Kamada et al. demonstrated that the presence of actively proliferating PD-1+ effector T-reg cells in tumors is a biomarker for HPD^32^. Kumagai et al. reported that lactic acid in the highly glycolytic TME, such as the liver, could induce active PD-1 + T-reg cells, leading to HPD^33^. Moreover, Li et al. proposed that various interactions via the IFNγ-FGF2-β-catenin axis are a feasible mechanism for causing HPD and could be a therapeutic target^34^.

Although definitions of HPD vary substantially across studies, TGR (tumor growth rate), defined as the percentage increase in tumor volume per month, and TGK (tumor growth kinetics), defined as the change in tumor size per unit of time, have been commonly utilized for HPD diagnosis. Park et al. systematically reviewed 24 studies involving 3109 patients to assess HPD definitions^35^. They reported that 20 out of 24 studies employed the calculation of tumor growth acceleration (TGR ratio and/or TGK ratio) to define HPD. Calculating TGR or TGK ratios typically requires at least 3 radiologic examinations (pre-baseline, baseline, and post-treatment), whereas 2.1–39.1% of cases were excluded in eight studies due to missing imaging studies. In our case, although a pre-baseline assessment was performed 40 days before the initiation of avelumab, resulting in continuing CR, a post-treatment PET/CT scan to calculate the TGR or TGK ratio could not be evaluated due to the lack of a baseline radiographic assessment. The lack of an exact baseline assessment at the initiation of avelumab prevented us from assessing the possibility of progression before starting avelumab. Since the patient had no signs or symptoms during the 40 days prior to avelumab initiation, we did not conduct a radiographic examination as the baseline assessment of the extent of the disease. Nonetheless, the rapid patient deterioration and the emergence of extensive metastases only three weeks after the initiation of avelumab could be indicative of HPD. The lack of a standardized HPD definition has led to reported frequencies ranging widely from 4% to 37.3%^36^, highlighting the need for refinement and standardization of HPD criteria to identify clinically relevant HPD.

In summary, we described a fatal case of HPD following maintenance avelumab treatment in an mUC patient. Omics analyses of the autopsy samples suggested the important role of M2 macrophages, TGF-β signaling, and IL-8 in the pathogenesis of HPD. Further study is warranted to explore the role of these markers in a larger and prospective cohort.

## Methods

### Use of patient information

The experimental designs were approved by the institutional review board at Osaka Medical and Pharmaceutical University [OMPU-IRB approval number: No. 2305-11 (approval date: Nov 24th, 2017), No. 2344-10 (approval date: Nov 24th, 2017), No. 2523-3 (approval date: Jul 27th, 2018), No. 2808-2 (approval date: Jan 10th, 2020), No. 2571-6 (approval date: Nov 7th, 2018)] and National Cancer Center Japan (NCC-IRB approval number: 2020–486, Date of approval: February 9th, 2021). Consent of collecting tumor samples and peripheral blood was obtained directly from the patient. Consent of immediate autopsy was obtained from the family and is maintained on file at the Osaka Medical and Pharmaceutical University.

### Tissue specimen review

Primary and metastatic tumors were taken by the tumor resection with transurethral resection and autopsy respectively both of which were conducted at Osaka Medical and Pharmaceutical University hospital and were immediately stored in the RNAlater reagent (Thermo Fisher Scientific). Hematoxylin and Eosin (H&E) stained specimen underwent a rigorous review by a board-certified pathologist to ascertain its histological consistency with BC. The extraction of nucleic acids was facilitated using the DNA/RNA AllPrep kit (QIAGEN). We employed the NanoDrop Microvolume UV–Vis Spectrophotometer (Thermo Fisher Scientific) for the precise quantification of nucleic acids. Furthermore, RNA integrity was assessed using the Agilent 2100 Bioanalyzer (Agilent) to obtain an RNA Integrity Number (RIN); primary tumor presenting a RIN over 7.0.

### WES and RNA-seq

In this study, all the procedures for the library preparation were handled by Maholo LabDroids (humanoid robotic crowd biology) at Robotic Biology Institute Inc. (Tokyo, Japan)^37^. With regard to the WES and RNA-seq library preparation, all processes were conducted as previously described^9^. All WES and RNA sequencing were performed on the Illumina NovaSeq 6000 platform to ensure a minimum depth of 200 × for WES and 50 million base reads for RNA sequencing.

### Bioinformatics analysis

All the procedures for bioinformatics analysis were performed as previously reported^9^. To comprehend the relative biological states across samples, the average z-score was calculated for each gene set of HALLMARK. Initially, the z-score for each gene was computed from the RNA expression data, which had been normalized to Transcripts Per Million (TPM), for all samples from the OMPU-NCC cohort. Then, the average z-score was calculated from the z-scores of the genes included in each gene set. CNVkit^38^ was employed for the analysis of copy number variations. This software implements the methods for analyzing copy number variations using target sequences. Based on the region data used for WES mapping, the read depth of both on-target and off-target regions was utilized to improve the quality of estimation. The WES date from peripheral blood cells was used as a normal control, and the copy number of the primary and each metastatic tumor was individually estimated by the software.

### Immunohistochemistry

After transurethral resection and autopsy, primary and metastatic tissue samples were immediately fixed in 10% neutral buffered formalin for 12–24 h to manufacture formalin-fixed paraffin-embedded (FFPE). Immunohistochemical (IHC) staining was conducted using the Discovery ULTRA System (Roche Diagnostics, Basel, Switzerland) as per the manufacturer’s guidelines. The panel of antibodies was employed to evaluated protein expression, including CD8 (mouse monoclonal, C8/144B, DAKO), CD163 (mouse monoclonal, 10D6, Leica), FOXP3 (mouse monoclonal clone, 236 A/E7, Abcam) and IL-8 (rabbit polyclonal, 17038-1-AP, Proteintech). At least two pathologists independently assessed the immunohistochemistry results to ensure accuracy and reproducibility.

### Multiple immunofluorescence

We employed Opal™ 4-Color Manual IHC Kit (Akoya Biosciences) for multiple immunofluorescence. The protocol followed the manufacturer’s guidelines. To evaluate the ratio of CD163/CD68-positive cells, pan-macrophages and M2-macrophages were stained using CD68 antibody (red: mouse monoclonal, KP1, DAKO) and CD163 antibody (green: mouse monoclonal, 10D6, Leica), respectively. To identify cells in which IL-8 is produced, pan AE1/AE3 antibody (green: mouse monoclonal, AE1/AE3, Nichirei) and CXCL8/IL-8 antibody (red: rabbit polyclonal, 17038-1-AP, Proteintech) were used. At least two pathologists independently assessed the immunohistochemistry results to ensure accuracy and reproducibility.

### ELISA array

To quantitate the cytokine levels in plasma of avelumab treated three patients (paired pre and post treatment), we used ProcartaPlex immune assay (Thermo Fisher Scientific) and evaluated 19 cytokines (listed in Supplementary Data 1). The ProcartaPlex 96-well plate and the plasma samples were prepared according to the manufacturer’s instructions. Each of the samples was analyzed in duplicate and Varioskan LUX (Thermo Fisher Scientific) was used for the detection and quantification of the cytokines. When the signals were below the limit of detection, they were considered zero.

## Supplementary information

Title and legend of Supplementary Data 1 and 2

Supplementary Data 1

Supplementary Data 2

## Data Availability

The raw sequencing data and clinical data can be obtained from the Osaka Medical and Pharmaceutical University Translational Research Program Biobank (OMPU-TR Biobank) (https://www.ompu.ac.jp/department/rdcenter/transregular/); however, restrictions apply to the availability of these data, and they are not publicly accessible. Data are available to National Cancer Center Research Institute and OMPU investigators and their external affiliates, including academic and commercial partners, provided that they have approval from the Institutional Review Board (IRB) and a data use agreement. Samples and data shared with external entities must be de-identified. Any additional information required to reanalyze the data reported in this work is available from the Lead Contact (KK; kazumasa.komura@ompu.ac.jp, AY; ayoshimi@ncc.go.jp) upon request.
