# Supplementary material for: Mechanistic insights into lethal hyper progressive disease induced by PD-L1 inhibitor in metastatic urothelial carcinoma
Source: NPJ Precis Oncol. 2024 Sep 17;8:206. doi: 10.1038/s41698-024-00707-6 (PMC11408499; doi:10.1038/s41698-024-00707-6)
Supplement: Supplementary file 1 — Title and legend of Supplementary Data 1 and 2 [file 41698_2024_707_MOESM1_ESM.pdf]

Title

Supplementary Data 1: TGF-beta signaling was the most activated gene set in metastatic specimens.

Supplementary Data 2: Plasma IL-8 level chronologically increased in the HPD case.

Legend

Supplementary Data 1: The z-scores of hallmark gene sets in primary and metastatic tumors.

Supplementary Data 2: Each value of ELISA analysis of 19 cytokines on plasma samples from three patients treated with avelumab (this case and two avelumab responders).
